# Impact of fatigue at the shoulder on the contralateral upper limb kinematics and performance

**DOI:** 10.1371/journal.pone.0266370

**Published:** 2022-04-01

**Authors:** Frédérique Dupuis, Gisela Sole, Catherine Mercier, Jean-Sébastien Roy

**Affiliations:** 1 Faculty of Medicine, Université Laval, Quebec City, Canada; 2 Centre for Interdisciplinary Research in Rehabilitation and Social Integration, Quebec City, Canada; 3 Centre for Health, Activity and Rehabilitation Research, School of Physiotherapy, University of Otago, Dunedin, New Zealand; Toronto Rehabilitation Institute - UHN, CANADA

## Abstract

**Background:**

Altered movement patterns have been proposed as an etiological factor for the development of musculoskeletal pain. Fatigue influences upper limb kinematics and movement performance which could extend to the contralateral limb and potentially increasing risk of injury. The aim of this study was to investigate the impact of fatigue at the dominant arm on the contralateral upper limb movement.

**Methods:**

Forty participants were randomly assigned to one of two groups: Control or Fatigue Group. All participants completed a reaching task at the baseline and post-experimental phase, during which they reached four targets with their non-dominant arm in a virtual reality environment. Following the baseline phase, the Fatigue Group completed a shoulder fatigue protocol with their dominant arm only, while the Control Group took a 10-minute break. Thereafter, the reaching task was repeated. Upper limb and trunk kinematics (joint angles and excursions), spatiotemporal (speed and accuracy) and surface electromyographic (sEMG) activity (sEMG signal mean epoch amplitude and median frequency of the EMG power spectrum) were collected. Two-way repeated-measures ANOVA were performed to determine the effects of Time, Group and of the interaction between these factors.

**Results:**

There was a significant Time x Group interaction for sternoclavicular elevation range of motion (*p* = 0.040), movement speed (*p* = 0.043) and accuracy (*p* = 0.033). The Fatigue group showed higher contralateral sternoclavicular elevation and increased movement error while experiencing fatigue in the dominant arm. Moreover, the Control group increased their speed during the Post-experimental phase compared to baseline (*p* = 0.043), while the Fatigue group did not show any speed improvement. There was no EMG sign of fatigue in any of the muscles evaluated.

**Conclusion:**

This study showed that fatigue at the dominant shoulder impacts movement at the contralateral upper limb. Such changes may be a risk factor for the development of shoulder pain in both the fatigued and non-fatigued limbs.

## Introduction

Repetitive execution of altered movements (i.e., in terms of muscle activity and kinematic) has been proposed as an etiological factor for the development and maintenance of musculoskeletal pain as it increases the mechanical loads on musculoskeletal periarticular structures such as tendons and muscles [1–3]. The shoulder, the most mobile joint of the body, relies on its fine-tuning stabilizing muscles for its stability, especially in elevated arm positions. The shoulder is therefore particularly at risk of injury in the presence of altered movements [1,4,5]. In fact, movement alterations at the shoulder and scapula are regularly observed in injured populations [2,4–7].

Attempts have been made to understand why these movement alterations occur. We have previously shown that the presence of fatigue after performing a shoulder elevated task in healthy individuals has an impact on the kinematics of the trunk and upper limb and on motor performance during reaching movements [8,9]. Some of the observed alterations were similar to those observed in injured population, including greater use of clavicular elevation and lesser use of glenohumeral elevation [10]. Other studies have also shown similar upper limb adaptations in a state of fatigue during hammering [11], ratcheting [12] and chain work [13–15]. Thus, the kinematic changes observed in the presence of fatigue are potentially risk factors for developing and maintaining shoulder pain [8,16–18].

Various physiological processes contribute to fatigue, usually categorized into two domains: central and peripheral fatigue [19]. Peripheral fatigue refers to the physiological processes that influence contractile function, while central fatigue refers to the failure of the central nervous system (CNS) to drive the motoneurons adequately [20]. Central fatigue has been shown to result in delayed muscle activation [21] and muscle force variability [22–24]. It has also been reported to have cross-over effects, which means that performing a fatiguing task in one limb could lead to contralateral limb adaptations. For example, performing a fatiguing task with one leg has been shown to lead to decreased electromyographic (EMG) maximal voluntary contraction (MVC) of the contralateral limb [25–28]. This phenomenon has also been observed at the upper limb where inducing fatigue in the first dorsal interosseous decreased the MVC of the contralateral homologous muscle [29]. While central mechanisms of fatigue are known to impact contra-lateral muscle maximal activity, to our knowledge, no study has yet investigated how they influence the kinematics and performance of the contralateral limb. As motor adaptations are thought to increase injury risks [8], it is essential to understand the effect of the central mechanisms of fatigue in the contra-lateral limb kinematics.

The primary objective of this study was to investigate whether performing a shoulder fatiguing task in the dominant arm influences upper limb kinematics and performance (speed, accuracy, reaction time) of the non-dominant arm. Although there is limited evidence available, the hypothesis was that central mechanisms of fatigue would lead to similar movement alterations as observed in the dominant arm, i.e., an increase in trunk and sternoclavicular joint movements, reductions in glenohumeral joint movement and reduction of movement speed and accuracy [8,9]. We previously demonstrated that the fatigue protocol used in the present study leads to EMG signs of fatigue in the anterior and middle deltoid while performing the same reaching task (decreased median power frequency and increased EMG amplitude) [8]. The secondary objective of study was therefore to assess whether there are cross-over effects on muscle fatigue as well.

## Methods

### Participants

Healthy adults aged between 18 and 40 years with no upper limb or neck pain or limitations (full shoulder and neck active range of motion) were recruited. They were excluded if they had: 1) previous neck and upper limb surgery or fracture; 2) history of glenohumeral dislocation. They were recruited through the institutional mailing list of *Université Laval* and through social media. The Ethics Committee of the CIUSSS-CN (Rehabilitation and Social Integration section) approved this study and all subjects provided informed written consent.

### Study design

Participants took part in one laboratory session where they first completed a questionnaire on sociodemographics and the Edinburgh Handedness Inventory to establish hand dominance. Using a randomized list stratified by sex [26], participants were randomly assigned to either the Control group or the Fatigue group. All participants completed the two phases of the experiment as follow:

- Baseline phase: participants performed a unilateral reaching task with their non-dominant arm.
- Post-experimental phase: participants performed the same unilateral reaching task with their non-dominant arm. Participants in the Fatigue group completed the task following a validated experimental shoulder fatigue protocol with their dominant arm [16] while participants in the Control group completed the task after a 10-minute break (similar to the average time it took to perform the fatigue protocol).

### Reaching task

A virtual reality environment was used to create a standardized, individualized elevated reaching task. The virtual reality environment is a perfect tool to place targets around the participants in their 3D space and relative to their anthropometric characteristics (arm length, height, etc.). Every participant had a period of familiarization with the virtual reality environment, so they were comfortable and comfortable with it before the beginning of the task.

This virtual task, used in previous research by Dupuis et al. [9], consisted of a series of four virtual targets (5 cm radius balls) that participants had to reach from a standardized initial position. Participants wore HTC VIVE goggles with 3D depth information (HTC corporation, VIVEPORT, Taoyuan City, Taoyuan County, Taiwan) and held a controller in their dominant hand that appeared to them as a virtual hand in the virtual reality environment. They could see a 3 cm radius circle in the center of their virtual palm. They were instructed to reach the targets by placing the circle in their virtual palm directly on the 5 cm radius targets. The virtual task was created in Unreal Engine (Epic games international, Unreal Engine, Switzerland).

The starting position was standardized at 90° shoulder flexion, extended elbow, and neutral humeral rotation, called the Initial target position (ITP). Participants were seated on a stable chair without a backrest so that the trunk could move freely. The initial posture was standardized: knees flexed at 90° and feet on the floor. To begin the phase, they placed their virtual hand on the ITP for two seconds to release the first target. They were then instructed to reach that target as fast and accurately as possible, using the most direct path. Once reached, the target disappeared, and the participants had to return to the ITP to release the next one. Each of the four targets appeared five times in random order, for a total of 20 targets reached to complete the phase. When completing the task, participants were asked to move naturally without moving their feet. They had to rate their perceived level of exertion before and after each of the two phases.

As previously used by Dupuis et al. [8], four targets positions were chosen to evaluate the impact of fatigue in various plane of arm elevation as follow:

- Target 1: 90° humeral abduction (ABD), elbow extended.
- Target 2: humeral ABD + 90° external rotation (ER), 90° flexed elbow.
- Target 3: 120° humeral elevation in the scapular plane, extended elbow, neutral humeral rotation.
- Target 4: 120° humeral elevation in the sagittal plane, extended elbow, neutral humeral rotation.

The targets were positioned in the virtual reality environment for each participant using an electronic goniometer prior to the experiment.

### Fatigue protocol

Immediately after the Baseline phase, participants in the Fatigue Group performed a shoulder fatigue protocol with their dominant arm only. The fatigue protocol used in this study has previously been validated and included three activities: 1) manipulating screws on a wooden board for 2 minutes with the shoulders at 45° of flexion and with the elbows fully extended; 2) 20 repetitions of arm elevations in the sagittal plane holding a dumbbell (2 pounds for women; 4 pounds for men); and 3) 20 repetitions of arm elevations in the scapular plane holding the same dumbbell [10,16]. These three activities were repeated until the participants reached at least 8/10 of perceived exertion at the dominant shoulder, rated every 30 seconds using the Borg scale perceived level of exertion. The post-experimental phase was then performed immediately after finishing the fatigue protocol.

### Instrumentation and data analysis

#### Kinematics

Kinematic variables included trunk, scapular, shoulder and elbow joints movement, measured using six inertial measurement units (IMUs) (MVN, Xsens Technologies, Enschede, Netherlands; sampling rate: 60 Hz). The sensors were placed on the head, sternum, pelvis, scapula, upper arm and forearm of the participant’s non-dominant arm in accordance with Xsens suggested sensors configuration using Velcro straps [30]. Anthropometric measures (height, shoulder width, arm span, hip height, hip width, knee height, ankle height, foot size and sole height) were recorded and entered in the MVN Studio software (MVN studio software, v. 4.4.0, Xsens Technologies, Enschede, Netherlands). The Npose + walk calibration was used to calibrate the Xsens system as recommended by Xsens.

The mean values for kinematic variables were calculated for each target. Joint angles at the non-dominant arm were computed from the kinematic data using MVN studio and Xsens modified ISB body segment model. Kinematics of the trunk (lateral flexion [LF], rotation and flexion/extension), sternoclavicular joint (elevation), elbow (flexion-extension) and glenohumeral joint (flexion, ABD and ER) were assessed. There were three kinematics variables of interest. First, 1) the initial joint angles described as joint angles while waiting on the ITP between the reaching movements. This variable reflects the initial posture of the participants while waiting to begin the reaching. The two other variables were 2) the final joint angles (angles when the targets were reach) and 3) the total joint angular excursions (final angle–initial angle). These two variables reflect the reaching strategy as they characterize the reaching movement. Trunk lateral flexion, rotation, and the glenohumeral plane of movement were assessed when reaching toward targets 1, 2 and 3; glenohumeral rotation was assessed for movements toward target 2; all remaining movements were assessed across all the four targets.

#### Performance

Spatiotemporal data were measured using the controller held in the non-dominant hand and Unreal Engine during the task (sampling rate: 90 Hz) [31].

Spatiotemporal outcomes were extracted using Unreal Engine and using a custom software written in MATLAB [8]. The mean values of the following variables were calculated to characterize participants’ performance:

- The reaction time: time from the moment the target was released from the ITP and the moment the participant initiated the reaching movement (i.e., the moment the hand quit the ITP).
- Movement speed: the time from the moment the participant initiated the reaching movement (quit the ITP) and the moment the target was reached.
- The initial angle of endpoint deviation (iANG): absolute angle calculated using the shortest line between two targets (the ITP and the reaching targets) and the line corresponding to the initial peak of acceleration, which reflected movement planning as it was based on the initial trajectory of the hand.
- The final error (fERR): the shortest arc distance between the ideal arrival point into the sphere (target) and the actual arrival point, which reflected the accuracy of the movement.
- The area under the curve: the summation of the rectangular trapezoids perpendicular to the ideal trajectory line and the actual trajectory line, which is the total movement error.

#### EMG analysis

For the secondary objective of this study, the presence of fatigue in the main agonist muscles of the task was assessed using three wireless surface EMG (sEMG) sensors (Delsys Trigno, USA) placed on the anterior and middle deltoids and the upper trapezius of the non-dominant arm [32]. The skin was cleaned using alcohol prior to electrode placement, the latter positioned according to Surface EMG for the Non-Invasive Assessment of Muscles (SENIAM) guidelines [33]. Muscle activity was recorded using Delsys EMGworks^®^ Acquisition software (sampling rate: 1925.93Hz).

The presence of fatigue in the three main agonist muscles was characterized as a downward shift in the EMG power spectrum (i.e. median power frequency [MDF]), associated with an increase in sEMG signal mean epoch amplitude, which has been proposed as good indicator of neuromuscular adaptation associated with fatigue [8]. All EMG signals were processed using custom software written in MATLAB R2013a (The MathWorks Inc., Natick, Massachusetts, United States). EMG signals were digitally filtered off-line with a zero-lag 4^th^ order Butterworth Filter (band-pass 20–450Hz) and the band-passed signals were rectified [8]. The band-passed signals were rectified, and their sEMG signal amplitude (root-mean-square envelope of the EMG signal) and MDF were first separated in 2-second epochs, and then averaged over periods of 60 seconds for statistical analysis. The power spectrum density was computed from the squared Fast-Fourier Transform [34]. The mean values of sEMG signal epoch amplitude and MDF of the first and last 30 seconds of each phase were used for statistical analysis [8].

The three systems (Xsens, EMG and Unreal) were time-synchronized using a custom trigger box.

### Statistical analyses

Independent t-tests and χ2 were used to compare baseline demographic data of the two groups. Spatiotemporal data and joint kinematics were compared using a three-way repeated-measures ANOVA to calculate the effect of Time (Baseline, Post-experimental), Group (Fatigue, Control) and Target (Target 1, 2, 3 and 4), as well as the interaction effect between Time and Group. The perceived levels of exertion before and after each phase were compared using a three-way repeated measures ANOVA to calculate the effect of Time (Baseline, Post-experimental), Task (pre-phase, post-phase [because of the physical demands of the task]) and Group (Fatigue, Control), and the interaction between these factors. EMG data were also compared using a three-way repeated measures ANOVA to calculate the effect of Time (Baseline, Post-experimental), Task (mean values of the first 30 sec, mean values of the last 30 sec of the phase) and Group (Fatigue, Control), and the interaction between these factors (proc GLM, SPSS 26).

Inherent post-hoc tests (with Bonferroni corrections) were conducted to detail interactions between factors. All statistical tests were conducted in IBM SPSS Statistics (IBM SPSS Statistics 26, IBM Corp., NY, USA) with a significance level set at 0.05.

## Results

Forty healthy participants were randomly assigned to one of the two groups (Control group n = 20; Fatigue group n = 20). There was no significant difference for any of the baseline characteristics between the groups (Table 1, p > 0.05).

**Table 1:** Participant’s baseline characteristics.

Characteristics	Fatigue Group n = 20	Control Group n = 20
Sex, female n, %	7 (35)	9 (45)
Height, cm	172.5±9.3	170.4±9.4
Weight, kg	72.9±11.4	66.1±11.8
Age, years	26±3	26±3
Dominance, right n, %	18 (90)	20 (100)
Sports^†^, %	12 (60)	10 (50)
Work^†^, %	12 (60)	9 (45)
**Mean perceived level of exertion**		
Before baseline	0	0
After baseline	3.3±1.6	3.6±1.5
Before Post EXP	0.6±0.6 *	0.3±0.4 *
After Post EXP	3.5±1.3	3.4±1.6

$\bar{X}$±SD = mean.

†: Participants were asked if they were practicing a sport or/and having a job with a physical requirement of the shoulder (yes/no).

Baseline = baseline phase; Post EXP = Post experimental phase.

Mean perceived level of exertion rated on the Borg rating scale.

* Significant Time x Group interaction (p = 0.046), the mean perceived level of exertion at the beginning of the Post-experimental phase was higher than at the beginning of the Baseline phase for the Fatigue Group (Time effect p<0.0001).

### Perceived level of exertion

Participants in the Fatigue group completed the fatigue protocol with their dominant arm in 6.5±2.0 minutes and reached a mean perceived level of exertion at the dominant shoulder of 9.0±0.6 on the Borg rating scale.

As for the non-dominant arm, both groups showed a significant increase of their perceived level of exertion during both phases (i.e., finishing the phase with a higher perceived level of exertion than at the beginning), but there was no significant between-group difference (Task effect *p*<0.0001, Task x Group *p* = 0.588) (Table 1). However, there was a significant Time x Group interaction (*p* = 0.046). The perceived level of exertion at the non-dominant shoulder reported by the Fatigue group at the beginning of the task was significantly higher during the Post-experimental phase than during the Baseline phase (Time effect *p*<0.0001). As for the Control group, they showed similar perceived level of exertion during both phases (Time effect *p* = 0.446).

### Kinematics

**Fig 1:**
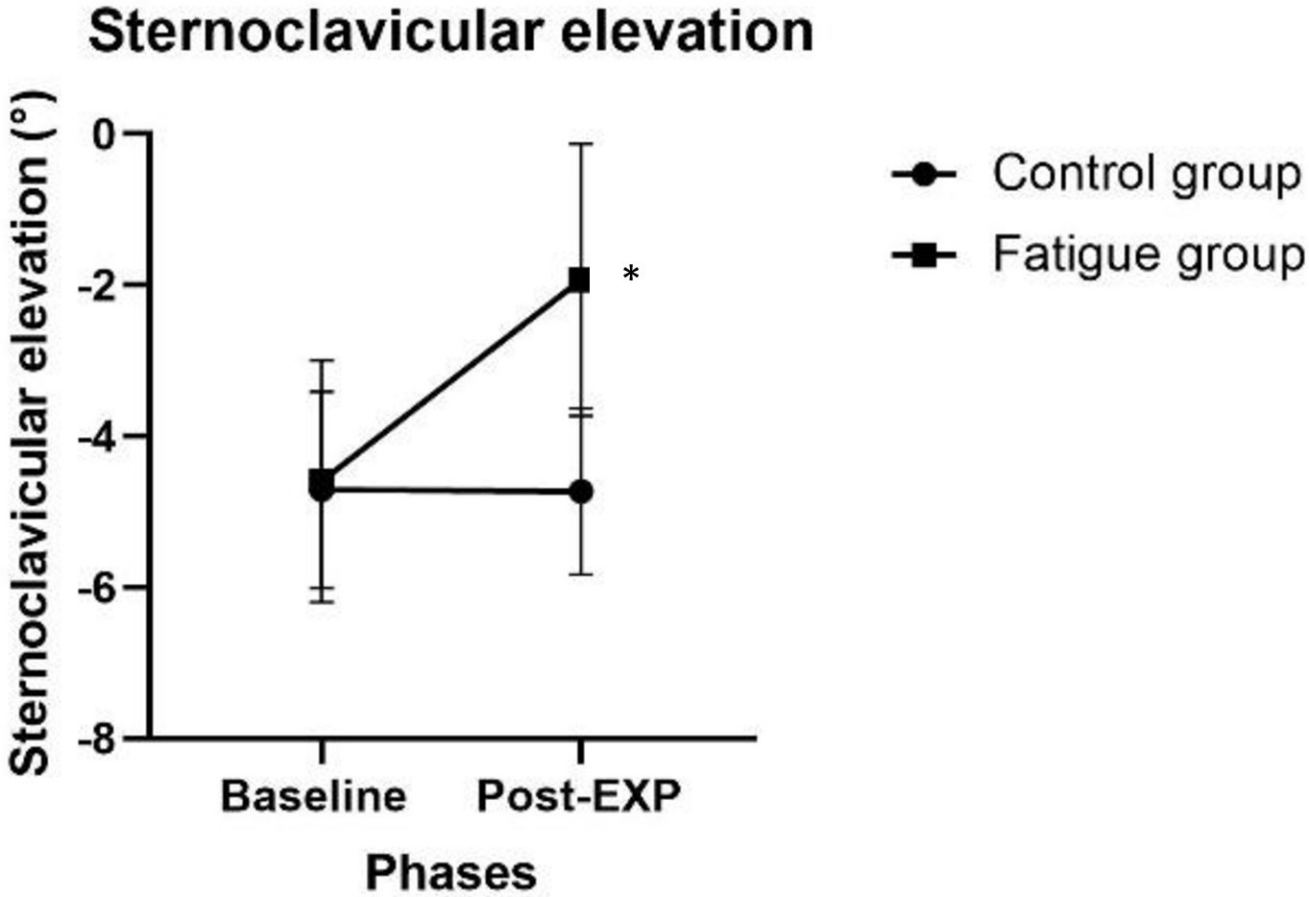
Sternoclavicular elevation initial position. Post EXP: Post-experimental phase. Results are presented as mean values for the 4 targets (SD) of the initial sternoclavicular elevation. Significant Time x Group interaction for the initial sternoclavicular elevation: The Fatigue group showed higher sternoclavicular elevation during the Post-experimental phase compared to the Baseline phase (*p* < .05).

There was a significant Time x Group interaction for the initial sternoclavicular elevation range of motion (*p* = 0.040, Fig 1). Post-hoc analysis showed that the Fatigue group had higher sternoclavicular elevation while waiting on the ITP during the Post-experimental phase compared to the Baseline phase (*p* = 0.017). There was no other significant interaction for kinematics data.

### Spatiotemporal variables

**Fig 2:**
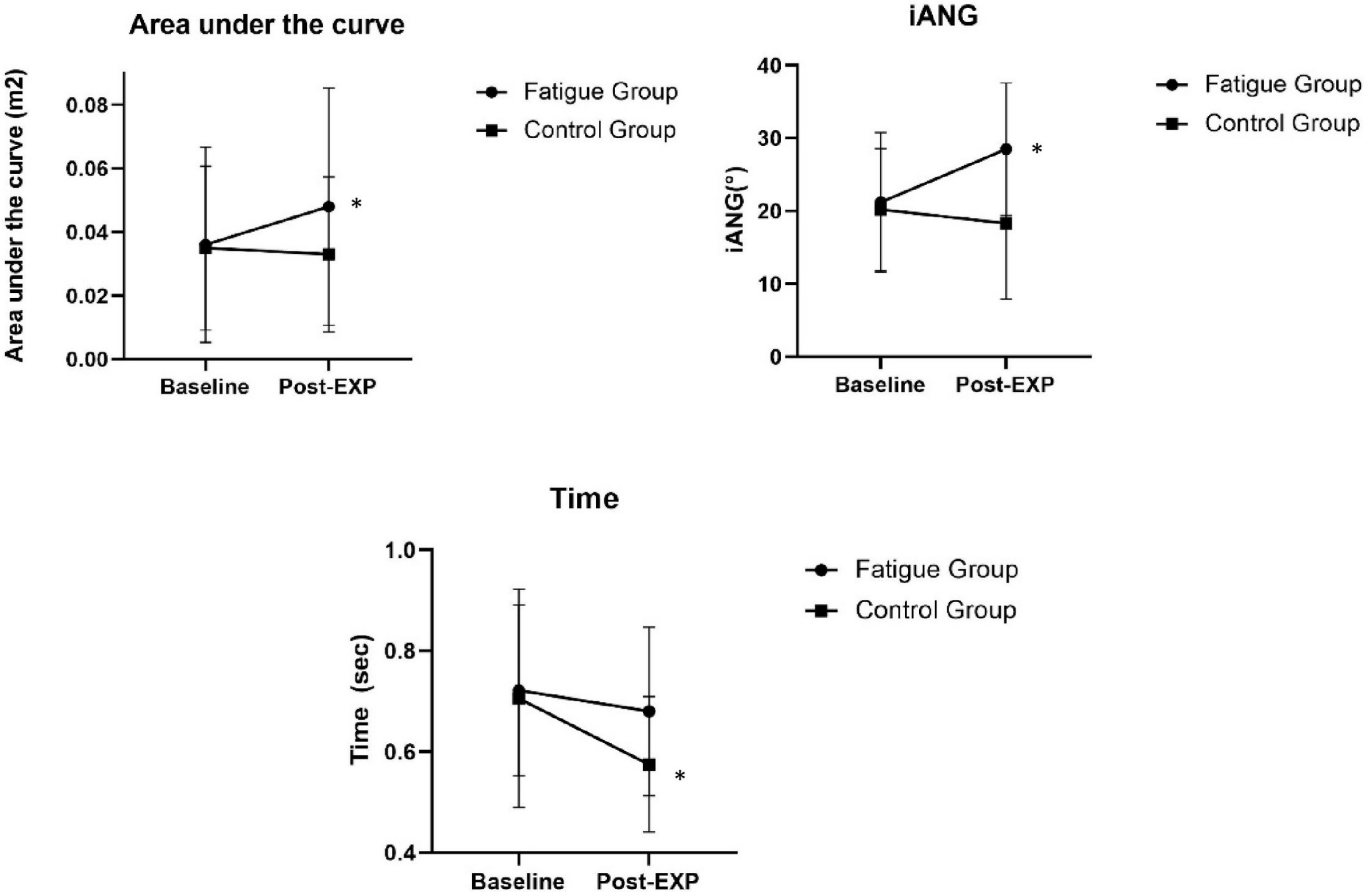
Spatiotemporal data. Post EXP: Post-experimental phase; Area: Area under the curve; iANG: Initial angle; Time: Time to reach the peak. Data are presented as mean values for the four targets (SD).

Significant Time x Group interaction were found for iANG (p = 0.005), Area under the curve (p = 0.033) and the mean Time to reach the target (p = 0.043). The Fatigue group increased their mean iANG (p = 0.007) as well as their mean Area under the curve (Fig 2, p = 0.008) while experiencing fatigue in the other shoulder during the Post-experimental phase. They also showed less accuracy during the Post-experimental phase, while the Control group did not showed any changes (Fig 2, p>0.215). In addition, the Control group increased their speed through the phases, significantly reducing their mean time to reach the target in the Post-experimental phase (p = 0.043). The Fatigue group did not show such a speed improvement (Fig 2, p = 0.145). There was no significant interaction for the reaction time and fERR (p>0.384).

### EMG data

There was a Time x Group interaction for the sEMG signal mean epoch amplitude of the Upper trapezius (*p* = 0.050). The Fatigue group showed an increase of sEMG signal mean epoch amplitude of the upper trapezius during the Post-experimental phase compared to baseline (*p* = 0.030) while the Control group did not show any changes (*p* = 0.244, upper trapezius sEMG signal mean epoch amplitude for Fatigue group at Baseline = 6.3±3.0^−5^ V; Post EXP = 7.1±3.2^−5^ V/ Control group at Baseline = 6.5±4.2^−5^ V, Post EXP = 6.7±4.3^−5^ V). There was no other interaction for EMG activity.

## Discussion

This study investigated the impact of fatigue at the dominant shoulder joint on kinematics and performance of the contralateral upper limb. The Fatigue group showed a greater use of contralateral sternoclavicular elevation and decreased performance while experiencing fatigue in the dominant shoulder. When performing the same reaching task, we previously demonstrated that the fatigue protocol used in the present study leads to sEMG sign of fatigue (decreased median power frequency and increased sEMG amplitude) in the main agonist muscles of the dominant arm (i.e., anterior deltoid and upper trapezius) [8]. In the present study, we aimed to investigate whether there are cross-over effects of muscle fatigue. We did not identify muscle sEMG signs for fatigue in the three main agonist muscles of the non-dominant arm, but a significant increase in upper trapezius sEMG signal mean epoch amplitude occurred in the Fatigue group during the Post-experimental phase.

### Kinematics

As hypothesized, the Fatigue group showed an increase of sternoclavicular elevation initial position (mean increase of 2.7±0.7°), reflecting a change in the initial posture following fatigue in the contralateral arm. We previously showed a similar scapular elevation increase (i.e., mean increase of 4.0±0.8°) while experiencing fatigue in the ipsilateral upper limb [8]. Increased sternoclavicular elevation has been observed in populations with shoulder pain and is thought to be a kinematic adaptation to protect the subacromial structure [2,5–7]. The central mechanisms contributing to the feeling of fatigue in the dominant arm could have also affected the contralateral arm movement planning, leading to similar protective kinematic adaptation in that (non-dominant) arm [1,2,4,5,8]. However, even if those fatigue-related movement planning changes are considered to be protective, they modify mechanical stress applied to the periarticular structures during movements (i.e., tendons, muscles, ligaments), which could be deleterious and lead to injuries.^1, 2^ Thus, it is reasonable to suggest that fatigue may increase risk of injuries in the fatigued limb as well as in the contralateral limb.

### Performance

As expected, the presence of fatigue in the contralateral side affected the performance of the non-dominant side. The Fatigue group showed greater movements errors during the Post-experimental phase (iANG and Area under the curve) and failed to improve their movement speed like the Control group. Altered proprioception has previously been observed in the contralateral limb when experiencing muscle fatigue and could explain the decreased movement accuracy during the Post-experimental phase in the Fatigue group [3,8,35]. For example, Sadler et al. reported a loss of proprioceptive estimation es accuracy in both hands after performing a pointing task until fatigue with the right hand only, suggesting that central effects of fatigue interfere with proprioceptive accuracy [3]. Altered central proprioceptive integration would also explain the lack of speed improvement as reliance on visual feedback becomes even more important to accurately perform the task, leading to a greater use of feedback controls [9]. An interesting aspect of performing reaching tasks in a virtual reality environment is that it would allow precise manipulation of visual feedback in future studies, as decreased visual feedback has been shown to alter reaching strategies and could therefore reveal the effect of proprioceptive deficits in a clearer manner [36]. Other factors may have contributed to the performance alterations. For example, divided attention has been shown to impair movement planning [37]. Thus, the higher perceived level of exertion that the Fatigue Group experienced in the non-dominant side at the beginning of the Post-experimental phase might have led to a reduced attention.

### EMG data

Using the same study design (i.e., task, fatigue protocol and analysis), we previously showed EMG signs of fatigue (anterior deltoid and upper trapezius) in the dominant arm of healthy subjects completing the task after performing the fatigue protocol [8]. Therefore, EMG analysis were conducted to investigate whether there were any cross-over effect of muscle fatigue. Despite the small, but significant, increased perceived level of exertion in the Fatigue group before the Post-experimental phase, there was no fatigue identified in the main agonists muscles during that phase. A decreased EMG maximal voluntary contraction has often been reported to be a cross-over effect of central fatigue [25–28]. However, maximal voluntary contractions are not usually performed in daily life tasks, which supports the relevance of studies investigating fatigue and cross-over effect during functional tasks (i.e., reaching). There was an increased upper trapezius sEMG signal mean epoch amplitude, and we hypothesize that this change could be explained by the protective kinematic adaptations that occurred at the sternoclavicular joint during the Post-experimental phase (i.e., increased sternoclavicular elevation). As the main agonist of sternoclavicular elevation, the upper trapezius showed an increase of its EMG signal amplitude, and as there was no decrease of the mean median power frequency, it supports the hypothesis that it was not caused by the presence of fatigue in the upper trapezius [38].

### Strength and limitations

To our knowledge, this study is the first to investigate contralateral upper limb kinematics adaptations in the presence of fatigue in the dominant shoulder. We previously used the same protocol to investigate the impact of fatigue in the dominant arm, which all together provides new evidence for the central mechanisms of fatigue and its clinical impact on movement and performance. The population in this study were young and healthy participants, which limits generalization. Therefore, exploring older or injured populations could lead to different results. Also, the perceived level of exertion in the dominant arm reached 9/10 in the Fatigue group. Such a high perceived level of exertion might not be representative of the fatigue experienced in real-life situations. We should therefore consider that the observed kinematics adaptations and performance alterations could differ from the present results in clinical populations.

## Conclusion

This study aimed to investigate the impact of fatigue on the contralateral upper limb movement. Participants in the Fatigue group showed higher sternoclavicular elevation during the Post-experimental phase with an increased upper trapezius sEMG signal mean epoch amplitude. Moreover, their movement accuracy was significantly reduced. This study showed that the central component of fatigue impacts on movement at the contralateral upper limb and could therefore be a risk factor for the development of shoulder pain in both the fatigued and non-fatigued limbs.
